# Impact of Audio-Visual Asynchrony on Lip-Reading Effects -Neuromagnetic and Psychophysical Study-

**DOI:** 10.1371/journal.pone.0168740

**Published:** 2016-12-28

**Authors:** Tetsuaki Kawase, Izumi Yahata, Akitake Kanno, Shuichi Sakamoto, Yoshitaka Takanashi, Shiho Takata, Nobukazu Nakasato, Ryuta Kawashima, Yukio Katori

**Affiliations:** 1 Department of Otolaryngology-Head and Neck Surgery, Tohoku University Graduate School of Medicine, Sendai, Miyagi, Japan; 2 Laboratory of Rehabilitative Auditory Science, Tohoku University Graduate School of Biomedical Engineering, Sendai, Miyagi, Japan; 3 Department of Audiology, Tohoku University Graduate School of Medicine, Sendai, Miyagi, Japan; 4 Department of Functional Brain Imaging, Institute of Development, Aging and Cancer, Tohoku University, Sendai, Miyagi, Japan; 5 Research Institute of Electrical Communication, Tohoku University, Sendai, Miyagi, Japan; 6 Department of Epileptology, Tohoku University Graduate School of Medicine, Sendai, Miyagi, Japan; Kyoto University, JAPAN

## Abstract

The effects of asynchrony between audio and visual (A/V) stimuli on the N100m responses of magnetoencephalography in the left hemisphere were compared with those on the psychophysical responses in 11 participants. The latency and amplitude of N100m were significantly shortened and reduced in the left hemisphere by the presentation of visual speech as long as the temporal asynchrony between A/V stimuli was within 100 ms, but were not significantly affected with audio lags of -500 and +500 ms. However, some small effects were still preserved on average with audio lags of 500 ms, suggesting similar asymmetry of the temporal window to that observed in psychophysical measurements, which tended to be more robust (wider) for audio lags; i.e., the pattern of visual-speech effects as a function of A/V lag observed in the N100m in the left hemisphere grossly resembled that in psychophysical measurements on average, although the individual responses were somewhat varied. The present results suggest that the basic configuration of the temporal window of visual effects on auditory-speech perception could be observed from the early auditory processing stage.

## Introduction

Visual speech information synchronously presented with speech sound can affect speech perception. Consequently, visual speech information, such as the speaker’s face uttering the speech sound, is important in the perceptual process of auditory input in individuals with impaired hearing, as perception of ambiguous speech sound can become clearer with the congruent visual speech information [1, 2]. This is commonly known as lip-reading. However, if the visual speech information is incongruent to the speech sounds, perception of the audio speech may be affected. For example, presentation of the /be/ sound (audio) with visual /ge/ (speaker’s face uttering the /ge/ sound), resulting in incongruent visual stimulus, often results in perception of the presented /be/ sound as the /de/ sound. This effect of incongruent visual speech information is well known as the McGurk effect [3].

Temporal synchronization between the audio and visual (A/V) stimuli is known to be critical for effective lip reading; i.e., there is a limited range referred to as the “temporal window” in which A/V perceptual binding occurs [4–7]. Based on psychophysical examination of the temporal window of McGurk phenomenon, high-rate fusion response (i.e., positive A/V integrative response) was observed in response to perfectly synchronous A/V stimuli (no lag) maintained over the temporal window of about 200–300 ms (i.e., +/-100 ms to +/-150 ms) with asymmetry between the audio vs. visual lags (more robust for audio lags) [4–7]. Greater temporal offset between the audio and visual stimuli caused lower fusion response whereas the asymmetry between audio and visual lags was maintained. That is, when audio stimuli lead visual stimuli by about 500 ms, no significant fusion response could usually be obtained. In contrast, when visual stimuli lead audio stimuli with the same offset, some fusion response could be observed.

Since simultaneous visual input influences auditory input in A/V speech perception, it would be important to know when, where, and how the visual inputs affect the processing of auditory inputs. The left superior temporal sulcus (STS) receives neural projections from both auditory and visual cortices, and is known to be important in A/V multimodal coupling [8–14]. In addition, speech processing in the auditory cortex, which is an earlier processing site than the STS in auditory signal processing, may be modulated by the visual effects conveyed via the direct corticocortical (visual cortex to auditory cortex) pathway, which does not involve the STS [15–18]. The visual effects on the auditory cortex can be observed in the auditory evoked N100 response of electroencephalography (EEG) and/or N100m response of magnetoencephalography (MEG) generated from around the auditory cortex [15]. The latencies of the N100(m) response to monosyllables are shortened and the amplitudes of those responses are decreased by the simultaneous presentation of visual speech information [12, 15, 19–24]. Moreover, as observed in the STS, dominant lip-reading effects in the left hemisphere also occur in these visual effects on the N100 response [12, 22]. On the other hand, the involvement of the right hemisphere in the processing of A/V coupling may complementally vary, based on the report of activation of the right hemisphere after total damage to the left temporal cortex [25].

Some predictive mechanism processing the preceding visual speech information is speculated to account for these facilitative speed-up and suppressive effects seen in the N100m caused by the presentation of visual speech information [19, 23, 24, 26]. However, whether a similar type of temporal window to that obtained in psychophysical A/V perception such as the McGurk effect could also be seen in this predictive visual effect on the N100m response has not been clarified. Considering that the temporal window appears to be one of the characteristics of the psychophysical A/V integrative phenomenon, what kind of temporal window could be observed in the auditory responses at the auditory cortex level with a latency of about 100 ms would be important to know to clarify the detailed mechanism of A/V coupling.

The present study examined the effects of asynchrony between audio and visual stimuli on the early auditory evoked fields (AEFs) using whole head MEG [27–36], focusing on the visual effects on the N100m originating from the left auditory cortex which is the dominant hemisphere in A/V coupling [8–14, 22, 37]. The N100m in response to the monosyllabic sound /be/ presented with a moving image uttering /ge/ (visual stimuli to cause McGurk effects) was assessed under 5 different A/V offset conditions (audio lag -500 ms, -100 ms, 0 ms [synchronous, no delay], +100 ms, and +500 ms), as well as the control condition (audio stimuli with visual noise), and the effects of A/V asynchrony on N100m responses, which were recorded from the left hemisphere, were compared with those on the psychophysical responses, which were recorded during measurement of the N100m.

## Methods

### Subjects

Eleven healthy volunteers (7 males and 4 females, mean age 30.7 years) without histories of auditory diseases and/or neurological disorders participated in this study. All subjects were native Japanese speakers and were classified as right-handed based on the Edinburgh Handedness Inventory (scores: above +90) [38]. All procedures of the present study were approved by the ethical committee of Tohoku University Graduate School of Medicine, and written informed consent in accordance with the requirements of the ethical committee was obtained from each subject. All parts of the present study were performed in accordance with the guidelines of the Declaration of Helsinki (1991).

### A/V stimuli

**Fig 1:**
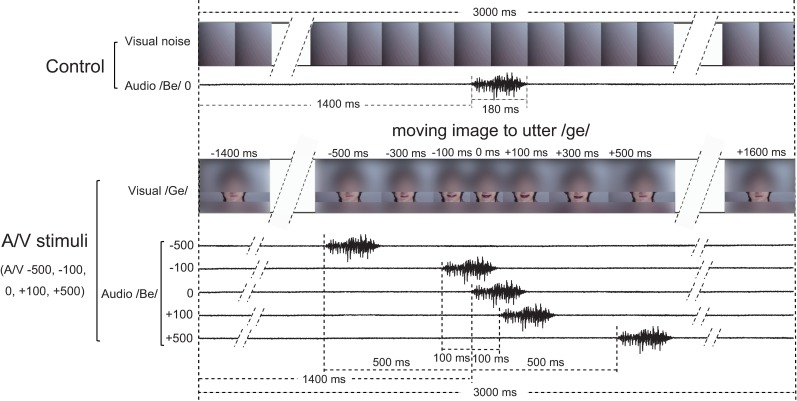
Audio-visual (A/V) stimuli used in the present study. Monosyllabic sound /be/ spoken by a Japanese male speaker was presented under six different visual conditions: 1) control: visual noise created by applying a strong Gaussian filter of a PC software (Adobe^®^ Photoshop) to a still image of the speaker’s face, and moving image of the same speaker’s face uttering /ge/ (incongruent visual stimuli known to evoke McGurk effect); 2) with synchronous conditions (no A/V lag) (A/V 0); 3) with +500 ms audio lag (A/V +500); 4) with +100 ms audio lag (A/V +100); 5) with -100 ms audio lag or +100 ms audio lead (A/V -100); and 6) with -500 ms audio lag or +500 ms audio lead (A/V -500). Each visual stimulus was prepared as a video clip with duration of 3 s. Audio stimulus started 1400 ms after the beginning of the visual stimulus and lasted for approximately 180 ms under the A/V 0 as well as control conditions (A/V noise). The presentation timing of audio stimuli were preceded or delayed 100 and 500 ms under A/V +/-100 and +/-500 ms conditions, respectively. The still images of visual stimuli at the timing points of -500 ms, -300 ms, -100 ms, 0 ms (at the onset of utterance), +100 ms, +300 ms, and +500 ms after the onset of utterance represent the moving images of uttering /ge/ (these face images in Fig 1 were treated with a digital filter except for the area around the mouth, so that the individual can not be identified). The movement of the speaker’s mouth started just before the time point -300 ms (300 ms before the onset of utterance) and ended just after the time point +300 ms (300 ms after the onset of utterance).

AEFs in response to the monosyllabic sound /be/ spoken by a Japanese male speaker were examined under six different visual conditions (Fig 1): 1) control: visual noise created by applying a strong Gaussian filter of a PC software (Adobe^®^ Photoshop) to a still image of the speaker’s face, and moving image of the same speaker’s face uttering /ge/ (incongruent visual stimuli known to evoke the McGurk effect); 2) with synchronous condition (no A/V lag) (A/V 0); 3) with +500 ms audio lag (A/V +500); 4) with +100 ms audio lag (A/V +100); 5) with -100 ms audio lag or +100 ms audio lead (A/V -100); and 6) with -500 ms audio lag or +500 ms audio lead (A/V -500). Each visual stimulus was prepared as a video clip with duration of 3 s. Under the A/V 0 conditions, the audio stimulus (/be/ sound) was presented instead of the original /ge/ sound of the visual stimulus, at the same timing as the original audio stimulus. Actually the audio stimulus started 1400 ms after the beginning of the visual stimulus and lasted for approximately 180 ms under the A/V 0 as well as control conditions (A/V noise). The presentation timing of audio stimuli were preceded or delayed 100 and 500 ms under the A/V +/-100 ms and +/-500 ms conditions, respectively. The movement of the speaker’s mouth started just before the time point -300 ms (300 ms before the onset of utterance) and ended just after the time point +300 ms (300 ms after the onset of utterance) as shown in Fig 1.

These six A/V stimuli were presented serially in random order using the software Presentation^®^ (Neurobehavioral Systems, Inc., Berkeley, CA). During the inter-stimulus interval, a black screen with small red cross, positioned around the “mouth” position in the moving image of the speaker’s face, was presented for 3,000 ms. Visual stimuli were projected on a monitor screen placed at a distance of 35 cm in front of the participants. Audio stimuli were presented through tube earphones (ER-3A, Etymotic Research, Elk Grove Village, IL) binaurally at a sound pressure level of 80 dB.

Subjects were instructed to listen carefully to the presented speech sound while observing the speaker’s face on the monitor screen, and were requested to report their perception of the A/V stimulus using three response buttons during the inter-stimulus intervals: the first and second buttons for /de/ and /be/, and the third button for other auditory percepts. These response data were recorded together with the MEG recordings.

### MEG recording and analysis

AEFs were recorded using a 200-channel whole-head type axial gradiometer system (MEG Vision PQA160C, Yokogawa Electric, Musashino, Tokyo, Japan) in a magnetically shielded room. The detailed conditions of the MEG system used in the study were described previously [39]. Briefly, the sensors, which consisted of first-order axial gradiometers with a baseline of 50 mm, were arranged in a uniform array over a helmet-shaped surface at the bottom of the dewar vessel. Each coil of the gradiometers was 15.5 mm in diameter. The mean distance between the centers of two adjacent coils was 25 mm. The field sensitivity of the sensors (system noise) was 3 fT/Hz within the frequency range. The MEG signal was band-pass filtered between 0.16 and 100 Hz and sampled at 500 Hz in most cases (10 cases), although in one case, data were sampled at 1,000 Hz. All MEG signals were recorded serially and later analyzed using the built-in software in the MEG system (MEG Laboratory, Yokogawa Electric). Awake state was confirmed by real-time MEG monitoring of the occipital alpha rhythm. To obtain and compare AEFs of each stimulus condition, MEG data from 1,100 ms before to 1,500 ms after the audio-stimulus onset were extracted from serial whole recording data with respect to each A/V stimulus condition, and then averaged (base line: from 1,100 to 0 ms before the onset of audio stimulus). The averaged data were digitally filtered from 2.0 to 45.0 Hz (band-pass). The N100m response was visually identified as the first prominent peak at 80–140 ms after the onset, with the isofield map confirming downward current orientation. The location of the signal sources of N100m was verified using an equivalent current dipole model with the best-fit sphere for each subject’s head, and the sources of N100m under all conditions were verified as located in or around Heschl’s gyrus.

In the present study, the latency and amplitude of N100m of the averaged wave (root mean square [RMS] waves) of all channels in the left hemisphere were assessed, and the measurements for different six A/V stimuli were compared. Usually, in most subjects (9 subjects), about 100–130 data points could be averaged to obtain the N100m response to each A/V condition. On the other hand, in two subjects, 63–65 data points could be averaged due to a shortage of recording data, but clear averaged waves of AEFs (no problems in terms of S/N ratio) could be observed. Thus, the data obtained from the latter two subjects were also included.

Analysis of variance with SPSS software was used to evaluate significant differences in N100m peak latency and amplitude in each hemisphere. *P* < 0.05 was considered to be significant.

## Results

### Psychophysical data

**Fig 2:**
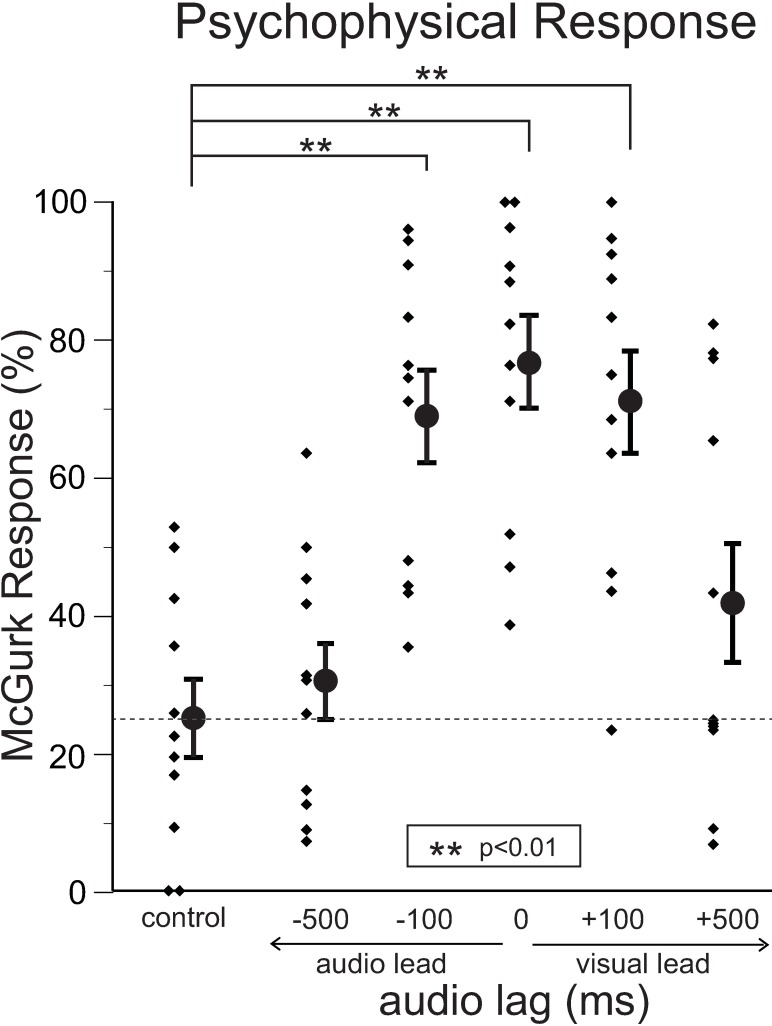
Psychophysical McGurk responses as a function of A/V offset. McGurk response rates are plotted with respect to each A/V condition. Small dots indicate individual data. Average and standard error values are represented by filled circles and bars, respectively. High fusion response rates were observed in response to synchronous A/V stimuli if the temporal asynchrony was within 100 ms. On the other hand, the fusion rate was remarkably reduced with temporal asynchrony of 500 ms, but the temporal window tended to be more robust (wider) for audio lags (asymmetry between audio vs. visual lags). Statistical significance of differences in positive fusion responses between each A/V offset condition and the control condition was determined by one-way repeated measures analysis of variance with Bonferroni post-hoc analysis. Asterisks indicate significant increase of fusion responses compared to the control condition.

The /be/ sound is often perceived differently (typically /de/ or /re/) due to the simultaneous presentation of visual speech /ge/ (McGurk effect) as a result of the A/V integrative fusion response. The positive McGurk response rate (perception other than /be/) of each subject was calculated based on the psychophysical response data recorded during the MEG measurements. McGurk response rates are plotted with respect to each A/V condition in Fig 2. Although the individual responses were somewhat varied, high fusion response rates (significantly positive A/V integrative response) were observed in response to synchronous A/V stimuli (no lag) as long as the temporal asynchrony was within 100 ms (A/V with -100 ms or +100 ms audio lag). In contrast, the fusion rate was remarkably reduced with temporal asynchrony of 500 ms (A/V with -500 ms or +500 ms audio lag), but the temporal window tended to be more robust (wider) for audio lags (asymmetry between audio vs. visual lags).

### Visual effects on N100m

**Fig 3:**
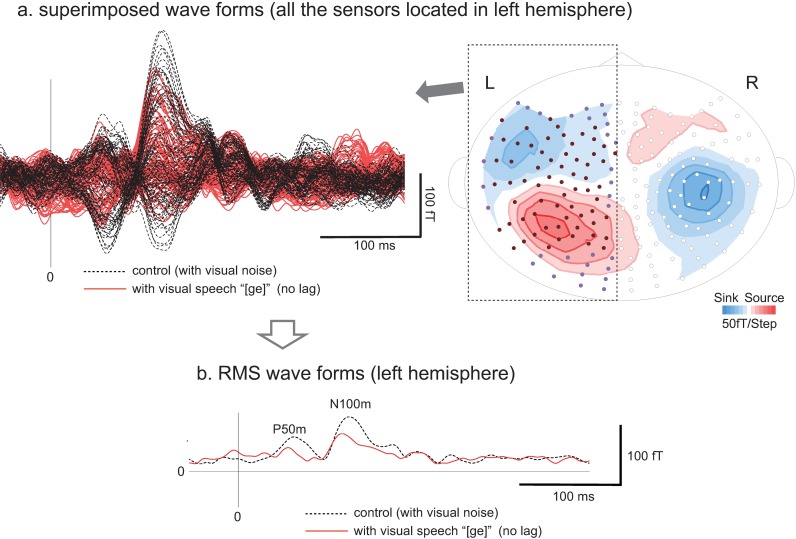
Typical example of the effects of visual speech (simultaneous [A/V offset = 0] condition) on the waveforms of AEFs observed in the left hemisphere. **a**: Superimposed waveforms recorded from all sensors located in the left hemisphere for control condition (/ge/ sound with visual noise: black dotted lines) and for A/V 0 condition (/ge/ sound presented with visual /be/ without no lag: red line). **b**: Root mean square (RMS) waveforms calculated from all sensors in the left hemisphere for control condition (/ge/ sound with visual noise: black dotted lines) and for A/V 0 condition (/ge/ sound presented with visual /be/ without no lag: red line).

Typical examples of the effects of visual speech (simultaneous [A/V offset = 0] condition) on the waveforms of AEFs observed in the left hemisphere are shown in Fig 3. Although the effects of visual speech could apparently be observed in the superimposed waveforms obtained from the sensors (Fig 3A), the average effects were assessed based on the averaged waves (RMS waves) of all channels in each hemisphere. The latency and amplitude of N100m were significantly shortened and reduced, as reported previously [12, 15, 19–24].

**Fig 4:**
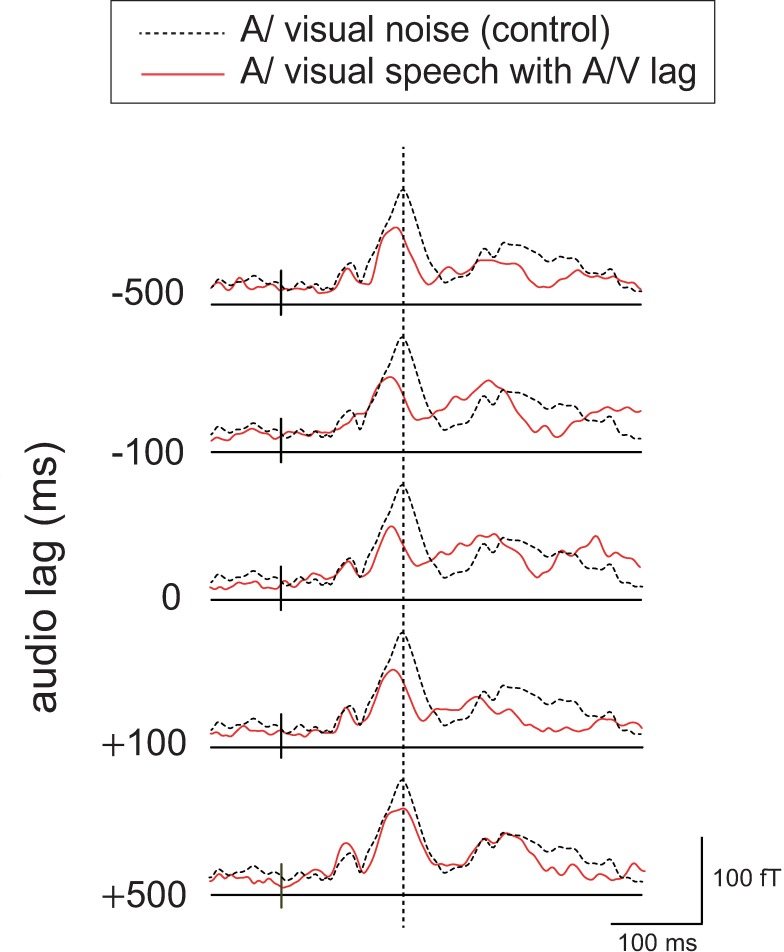
Typical examples of the effects of A/V lag on RMS waveforms of AEFs. Typical examples of the RMS waveforms are shown with respect to each A/V lag (AEFs under visual noise are indicated using black dotted lines), divided into each hemisphere (see "Visual effects on N100m" for further details).

**Fig 5:**
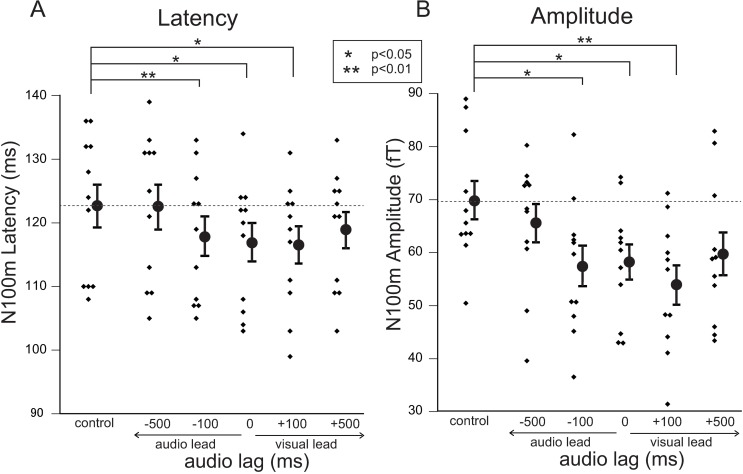
**Effects of visual speech on latency (A) and amplitude (B) of N100m in the left hemisphere.** Small dots indicate individual data. Average and standard error values are represented by filled circles and bars, respectively. In visual effects on both N100m latency and amplitude, significant effects (shortening of N100m latency and reduction of N100m amplitude) compared with the control condition (visual noise) are observed with temporal asynchrony within 100 ms. On the other hand, no significant effects could not be obtained with -500 ms audio lag. However, some effects (although not significant on average) were still observed in subjects with +500 ms audio lag on average. Statistical significance of differences was determined by one-way repeated measures analysis of variance with Bonferroni post-hoc analysis. Asterisks indicate significant differences.

Typical examples of the RMS waveforms with respect to each A/V lag are shown in Fig 4. The typical visual effects shown in the left hemisphere in Fig 3 (shortening of the latency of N100m with amplitude reduction) appeared to be almost preserved in the A/V lag within 100 ms. However, the latency shift caused by visual speech information was mostly reduced at +500 ms audio lag. In contrast, smaller effects appeared to persist with -500 ms audio lag. Total effects of visual speech on the latency and amplitude of N100m are shown in Fig 5.

Comparison of the effects on latency of N100m in the left hemisphere caused by visual speech stimuli under the five different conditions of audio lag (Fig 5A) showed that significant shortening of N100m latency compared with the control condition (visual noise) were observed with temporal asynchrony within 100 ms. On the other hand, almost no significant effects could be obtained with -500 ms of audio lag (visual lead) on average. However, some effects (although not significant on average) were still observed in temporal asynchrony with +500 ms audio lag; i.e., although the individual responses were also somewhat varied, a similar type of asymmetry of the temporal window as observed in the psychophysical results (Fig 2) was observed at least in the left hemisphere, again suggesting that the temporal window tended to be more robust (wider) for audio lags. Basically, similar trends were obtained in the effects on the amplitude of N100m (Fig 5B).

## Discussion

The present study examined the effects of A/V lag on the lip-reading effects observed in N100m of the left hemisphere in response to monosyllables using MEG, and the following effects were observed. The latency and amplitude of N100m were shortened and reduced by the lip-reading effects, as previously reported [12, 15, 19–24]. Similar levels of visual speech effects on N100m in response to synchronous A/V stimuli could be obtained with temporal asynchrony between audio and visual stimuli within 100 ms. In contrast, no significant latency shift was observed with 500 ms audio lag (visual lead) as well as 500 ms visual lag (audio lead) on average. However, some small effects were still preserved with audio lag of 500 ms on average, suggesting that similar asymmetry of the temporal window as observed in psychophysical measurements, which tended to be more robust (wider) for audio lags. These present results appear to be consistent with the idea that the basic configuration of the psychophysical temporal window for lip-reading (McGurk effect) can be observed at the level of the auditory cortex

The N100(m) response is known to be affected by visual information presented simultaneously, but the effects depend on the characteristics of the presented A/V stimuli. Using non-speech stimuli as the A/V stimuli (e.g., tone and non-speech visual cue), the amplitudes of N100(m) in response to tone stimuli could be increased if the tones were presented with the visual cues [26]. On the other hand, as shown in the present study, using A/V speech information as the A/V stimuli, shortened latency and amplitude reduction were observed in the auditory N100m responses by adding the visual speech information [12, 15, 19–24].

The underlying mechanism of the N100m amplitude reduction with shortened latency caused by the visual speech effects may involve some “predictive information conveyed by the preceding visual speech stimuli” via the direct fast pathway from the visual to auditory cortices (corticocortical pathway) [15–18, 20, 24]. That is, the preceding speech cue derived from the visual speech information (lip-reading information) may save and facilitate the auditory speech processing in the auditory cortices. The observation that the magnitude of visual speech effects on the N100m does not depend on the congruency between the audio and visual stimuli but is affected by the amount of syllable information transmitted only by visual speech information (without audio) agrees with this hypothesis [15]. The present results appear to show the presence of a temporal window in this predictive visual-speech effect on N100m. However, which factors in visual-speech stimuli are important and essential for the visual effects on N100m remain unclear. Further examinations using different visual stimuli, such as moving images to suggest the presentation timing of audio-stimuli and/or congruent visual-speech stimuli, etc., may provide additional useful information to clarify the detailed mechanism of the temporal window of visual effects on N100m.

In our experimental protocol, each experimental condition was presented in random order, and all recording data were serially saved in one file. Due to the limitations of the data memory in one file, a maximum of six experimental conditions could be examined at one time. Because of this limitation, the present experimental condition of A/V lag was too inaccurate to determine the precise temporal window. However, even considering these points, the present results basically indicate that there is a similar order of temporal window in the visual speech effects on auditory responses from the auditory cortices (which are supposed to be affected via the corticocortical pathway) to those observed in the psychophysical McGurk effect. This observation may imply that the psychophysical temporal window of A/V integration can be attributed to the temporal features of the predictive visual effects on auditory cortices, at least to some extent.

Auditory perceptions can be altered by incongruent visual speech information presented simultaneously in the McGurk effect. In typical experiments, the A/V speech stimuli generated by the same speaker are used to induce the McGurk effect. However, McGurk effects can be obtained even if there is a clear incompatibility in the speech characteristics between the audio and visual stimuli such as a male voice dubbed onto a female image, and McGurk effects of similar magnitude to those using the same speaker could be obtained [40]. That is, even though the participants notice the different origins of the A/V stimuli which provoke a feeling of inconsistency, the perceived speech sounds were clearly altered. Based on this well-known psychophysical feature of the McGurk effect, auditory signals may be affected by the voice characteristics extracted from visual speech information at an early stage of phonetic processing, prior to the integration of the A/V information [40].

Moreover, although temporal synchrony is one of the important factors to evoke A/V fusion response, the temporal window of the A/V fusion response may be wider than the temporal window of perception of synchrony (perceived simultaneity) [5]. That is, the McGurk effect could occur in the absence of perceived synchrony, and simultaneous judgment based on A/V information is not a prerequisite for positive visual effects. This evidence may also indicate that auditory speech signal is modulated at a relatively early phase of auditory processing. On the other hand, a more central region such as the multimodal center in the superior temporal cortex may be involved in the judgment of simultaneity.

Neurophysiological investigations suggest that the time series of speech processing in auditory system involves various processing stages according to the time elapsed from speech onset [41]. Acoustic-phonetic analysis starts by around 50–100 ms after stimulus onset and is typically reflected in the N100 response of EEG and N100m response of MEG [41–45]. Language-specific phonetic-phonological analysis subsequently follows, starting by 100–200 ms after stimulus onset. Any mismatch negativity in EEG or mismatch field in MEG is thought to be related to the events processed during this period [41, 46–48]. Superior temporal activation shows sensitivity lexical-semantic manipulation from about 200 ms onwards [41, 49–51].

Consequently, the present results (temporal characteristics of the visual speech effects on N100m) together with the above findings suggest that the visual speech effects impacting the phonetic analysis of the auditory signals occurred around the auditory cortices, which supposed to be early auditory processing with latency of 50–100 ms manifesting as the N100(m) response, may play some role in the McGurk effect. Moreover, the temporal characteristics of such visual speech effects, which are supposed to act via the direct corticocortical pathway from the visual cortex to the auditory cortex, may account for the basic features of the psychophysical temporal window of the McGurk effect as represented by the phonetic alteration of perceived speech sound.

## Supporting Information

S1 Data File containing the data used to replicate Fig 2 and Fig 5. (PDF)
