# Supplementary material for: Impact of Audio-Visual Asynchrony on Lip-Reading Effects -Neuromagnetic and Psychophysical Study-
Source: PLoS One. 2016 Dec 28;11(12):e0168740. doi: 10.1371/journal.pone.0168740 (PMC5193434; doi:10.1371/journal.pone.0168740)
Supplement: S1 Data — (PDF) [file pone.0168740.s001.pdf]

Fig. 2 Raw data

McGurk Response (%)

	sub 1	sub 2	sub 3	sub 4	sub 5	sub 6	sub 7	sub 8	sub 9	sub 10	sub 11
control	17.02128	22.64151	42.59259	19.64286	50	52.94118	26	35.71429	0	9.433962	0
audio lag −500	12.76596	25.92593	45.45455	31.48148	50	63.63636	30.76923	41.81818	14.81481	9.090909	7.407407
audio lag −100	35.55556	76.36364	74.54545	96.07843	90.90909	83.33333	48.07692	43.39623	71.15385	44.44444	94.44444
audio lag 0	38.77551	90.74074	82.35294	100	88.46154	96.2963	47.16981	51.92308	71.15385	76.36364	100
audio lag +100	23.52941	83.33333	75	100	88.88889	94.73684	63.63636	43.63636	46.2963	68.51852	92.45283
audio lag +500	6.976744	24.07407	82.35294	77.35849	78.18182	65.45455	24.4898	43.39623	23.52941	25	9.259259

Fig. 5 Raw data

A. N100m Latency (ms)

	sub 1	sub 2	sub 3	sub 4	sub 5	sub 6	sub 7	sub 8	sub 9	sub 10	sub 11
control	124	132	108	136	136	110	128	110	132	122	110
audio lag −500	125	131	109	139	131	105	131	109	133	121	113
audio lag −100	119	127	107	133	131	107	123	105	123	113	108
audio lag 0	124	124	106	122	134	103	120	104	122	118	108
audio lag +100	123	123	109	131	125	111	121	103	119	117	99
audio lag +500	123	121	111	133	127	109	125	103	125	121	109

B. N100m Amplitude (fT)

	sub 1	sub 2	sub 3	sub 4	sub 5	sub 6	sub 7	sub 8	sub 9	sub 10	sub 11
control	63.48	65.6	67.95	83.01	87.44	89.01	50.48	63.63	63.62	61.41	71.56
audio lag −500	62.06	68.29	67.78	74.48	72.75	80.27	39.6	73.28	49.04	60.73	72.66
audio lag −100	36.56	50.74	63.36	61.88	82.28	62.43	48.02	59.74	45.19	50.69	70.22
audio lag 0	43.04	53.98	60.48	64.14	74.26	61.92	44.72	57.18	42.97	62.79	73.19
audio lag +100	31.43	41.11	56.86	58.69	71.22	68.65	48.31	48.23	44.13	59.99	63.14
audio lag +500	43.41	58.84	53.81	80.67	70.77	59.13	46.04	55.57	44.48	60.61	82.93
